# Nanocomposites-based targeted oral drug delivery systems with infliximab in a murine colitis model

**DOI:** 10.1186/s12951-020-00693-4

**Published:** 2020-09-15

**Authors:** Jung Min Kim, Da Hye Kim, Hyo Jeong Park, Hyun Woo Ma, I Seul Park, Mijeong Son, So Youn Ro, Seokmann Hong, Hyo Kyung Han, Soo Jeong Lim, Seung Won Kim, Jae Hee Cheon

**Affiliations:** 1 Department of Internal Medicine and Institute of Gastroenterology, Yonsei University College of Medicine, 50-1 Yonsei-ro, Seodaemun-gu, Seoul, 03722 Republic of Korea; 2 Severance Biomedical Science Institute, Yonsei University College of Medicine, Seoul, Republic of Korea; 3 Department of Integrated Bioscience and Biotechnology, Sejong University, 209 Neungdong‑ro, Gwangjin‑gu, Seoul, South Korea; 4 Brain Korea 21 PLUS Project for Medical Science, Yonsei University College of Medicine, 50-1 Yonsei-ro, Seodaemun-gu, Seoul, 03722 South Korea; 5 Department of Integrative Bioscience and Biotechnology, Institute of Anticancer Medicine Development, Sejong University, Seoul, 05006 Republic of Korea; 6 College of Pharmacy, Dongguk University-Seoul, Dongguk‑ro‑32, Ilsan‑donggu, Goyang, South Korea

**Keywords:** Inflammatory bowel disease, Infliximab, Nanocomposite carrier, Oral delivery system

## Abstract

**Background:**

Infliximab (IFX), a TNF-α blocking chimeric monoclonal antibody, induces clinical response and mucosal healing in patients with inflammatory bowel disease (IBD). However, systemic administration of this agent causes unwanted side effects. Oral delivery of antibody therapeutics might be an effective treatment strategy for IBD compared to intravenous administration.

**Results:**

All three carriers had a high encapsulation efficiency, narrow size distribution, and minimal systemic exposure. There was a higher interaction between nanocomposite carriers and monocytes compared to lymphocytes in the PBMC of IBD patients. Orally administered nanocomposite carriers targeted to inflamed colitis minimized systemic exposure. All IFX delivery formulations with nanocomposite carriers had a significantly less colitis-induced body weight loss, colon shortening and histomorphological score, compared to the DSS-treated group. AC-IFX-L and EAC-IFX-L groups showed significantly higher improvement of the disease activity index, compared to the DSS-treated group. In addition, AC-IFX-L and EAC-IFX-L alleviated pro-inflammatory cytokine expressions (*Tnfa*, *Il1b*, and *Il17*).

**Conclusion:**

We present orally administered antibody delivery systems which improved efficacy in murine colitis while reducing systemic exposure. These oral delivery systems suggest a promising therapeutic approach for treating IBD.
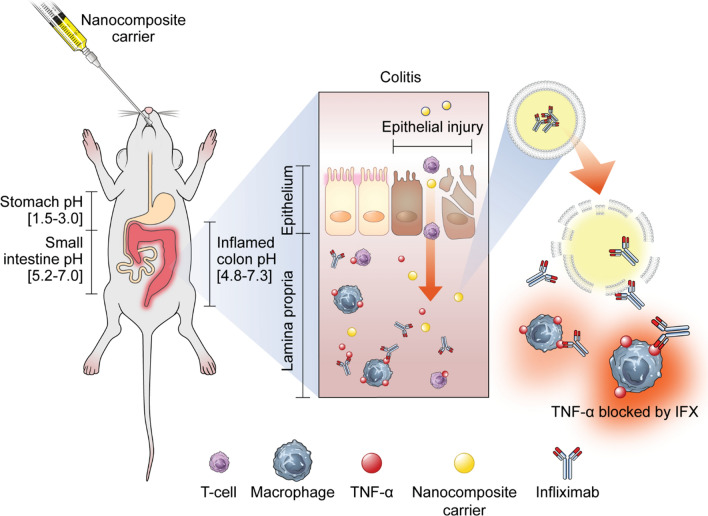

## Background

Inflammatory bowel disease (IBD) is a chronic relapsing inflammatory condition of the gastrointestinal tract. Cytokines are essential mediators of IBD pathophysiology and tumor necrosis factor (TNF)-α has a crucial function in the initiation and perpetuation of IBD [1]. TNF-α alters epithelial integrity, disrupts barrier function, and promotes the breakdown of intestinal homeostasis [2]. Moreover, the binding of TNF-α to the TNF receptor is associated with the prevention of apoptosis and prolongation of pro-inflammatory T cell survival in IBD [3]. Increased blood monocyte recruitment into the gut of IBD patients generates macrophages that leads to the secretion of inflammatory cytokines such as TNF, IL-6, and IL-23 [4].

Therapeutic monoclonal antibodies against TNF-α are used to treat IBD [5, 6]. These monoclonal antibodies have some advantages in terms of stability, bioavailability, and superior antigen targeting affinity. Infliximab (IFX) is a mouse/human chimeric monoclonal immunoglobulin G1 (IgG1) antibody against TNF-α. It directly neutralizes the biological activity of TNF-α. IFX is more effective at inducing clinical remission of IBD and mucosal healing than conventional drugs [7, 8]. However, IFX has infrequent but serious side effects such as infectious complications, autoimmune responses, and malignancy [9–11]. Furthermore, repeated intravenous administration of IFX is costly and associated with poor compliance [12]. Ideally, an anti-TNF antibody therapy for IBD would be delivered directly to the intestinal inflammatory sites, avoiding systemic exposure and immunosuppression.

Liposome is an attractive drug carrier; it has high biocompatibility and biodegradability, low toxicity, and non-immunogenic properties [13]. Liposomes are mainly composed of phospholipids, and the release rate depends on the number of phospholipid bilayers. Phospholipid barriers block the action of enzymes, acids, and free radicals, and protect the cargo from breaking down until it reaches the target site and is released. Specific pH range-triggered release and liposome encapsulation delays drug release specific to the gastrointestinal tract, which enhances therapeutic efficiency. Liposome-encapsulated IFX was observed to have therapeutic efficacy in autoimmune uveoretinitis [14]. In addition, liposomes provide better recovery of impaired epithelial barrier function than control in a dose-dependent manner [15]. Because ideal colon-specific delivery systems require the prevention of premature drug release, the properties of the liposome surface coating material encapsulating the anti-TNF-α agent are important because they affect the efficiency of the delivery system. Ideal colon-specific delivery systems require the prevention of premature drug release before reaching the target site.

Aminoclay is a synthesized organoclay 3-aminopropyl functionalized magnesium phyllosilicate. Oral delivery systems designed with aminoclay complex enhance the bioavailability of a low solubility/high permeability drug [16]. Indeed, aminoclay is less cytotoxic and can reduce cargo dosages, while enhancing delivery efficiency and allowing for the loading of macromolecules [17]. However, the therapeutic effect or improvement of impaired barrier function in IBD via aminoclay-anti-TNF-α complex has not yet been reported.

Eudragit (methacrylic acid copolymer) is a pH-sensitive coating polymer [18]. Eudragit with prednisolone was reported to efficiently degrade and deliver drugs only at colon-specific pH both in vivo and in vitro, thus minimizing drug side effects [19]. In addition, Eudragit was able to deliver 5-aminosalicylic acids to the target site with minimal systemic absorption [20]. Furthermore, the Eudragit coating system has a higher mucosa-adhesive ability than control coating systems [21]. However, no studies of Eudragit encapsulating anti-TNF-α have been reported in vivo or in vitro.

Therefore, the aim of the current study was to evaluate the effectiveness and safety of new oral colon-targeted IFX delivery systems using ternary nanocomposite carriers in a murine colitis model.

## Results

### Characterization and gastrointestinal stability of nanocomposite carriers

**Fig. 1:**
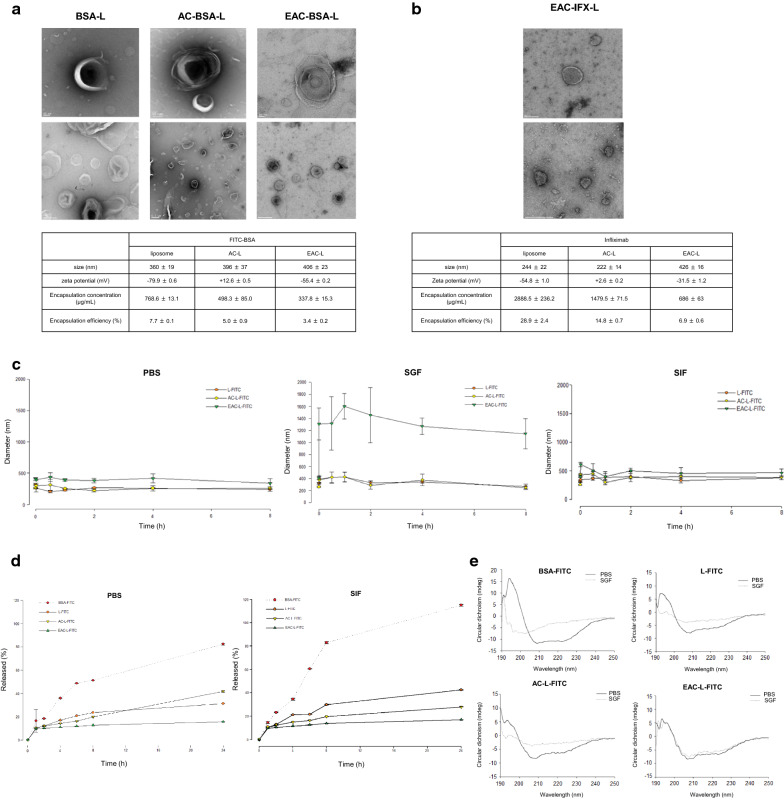
Characteristics of nanoparticles. **a** Transmission electron microscopy (TEM) images of fluorescein isothiocyanate (FITC)-bovine serum albumin (BSA) with nanocomposite carriers (liposome, aminoclay-coated, and Eudragit S100-clay-coated). Scale bar of single nanocomposite carrier represents 50 nm. The scale bar of BSA-L and EAC-BSA-L in the TEM images of lower magnification is 200 nm, and the scale bar of AC-BSA-L in the TEM images of lower magnification is 500 nm. The zeta potential is the mean ± SD of two independent experiments (*n* = 2), and the size and captured concentration data are the mean ± SD of three independent experiments (*n* = 2). **b** TEM images of Eudragit S100-aminoclay-coated liposomes entrapped with infliximab (IFX). Scale bar of single nanocomposite represents 100 nm. The scale bar of the TEM images of lower magnification is 200 nm. Size, zeta potential, and concentration of IFX encapsulated by nanocomposites were measured. AC-BSA-L, aminoclay-liposome-coated BSA; BSA, bovine serum albumin; BSA-L, liposome-coated BSA; EAC-IFX-L, Eudragit S100-liposome-coated IFX; Eudragit S100-aminoclay-liposome-coated IFX; FITC-BSA, BSA-fluorescein isothiocyanate conjugate. **c** Time-dependent size changes of liposome, clay-liposome, and E100-clay-liposome incubated in PBS, SIF, or SGF at 37 °C. **d** Time-dependent fluorescein isothiocyanate-bovine serum albumin (FITC-BSA) release profile from liposomes incubated in phosphate-buffered saline (PBS) or simulated intestinal fluid (SIF, without pancreatin) at 37 °C. **e** Circular dichroism analysis of the BSA stability in the nanocomposites

Transmission electron microscopy images showed that bovine serum albumin-fluorescein isothiocyanate (BSA-FITC) coated with liposome-coated BSA (BSA-liposome), Aminoclay-liposome-coated BSA (AC-BSA-L), and Eudragit S100-liposome-coated BSA (EAC-BSA-L) were spherical and surrounded the inner aqueous core containing BSA-FITC (Fig. 1a). Particle size and zeta potential analysis using dynamic light scattering showed a gradual increase in particle sizes (360, 396, and 406 nm in BSA-L, AC-BSA-L, and EAC-BSA-L, respectively) and reversal of zeta potential values (− 79.9, + 12.6, and − 55.4 mV in BSA-L, AC-BSA-L, and EAC-BSA-L, respectively) in the layer-by-layer coating. The net negative surface charge of BSA-L appeared to promote the alternating deposition of positively charged aminoclays through electrostatic interactions. The positively charged surface of AC-BSA-L allowed the deposition of anionic Eudragit S100, resulting in layer-by-layer coated EAC-BSA-L. Entrapped BSA-FITC concentrations of nanocomposites were 768.6 ± 13.1 μg/mL (BSA-L), 498.3 ± 85.0 μg/mL (AC-BSA-L), and 337.8 ± 15.3 μg/mL (EAC-BSA-L). A gradual decrease in entrapped protein concentration by coating was caused by the leakage of entrapped proteins during the layering process. Encapsulation efficiency were 7.7 ± 0.1% (BSA-L), 5.0 ± 0.9% (AC-BSA-L), and 3.4 ± 0.2% (EAC-BSA-L). Nano-sized IFX-entrapped liposomes gradually increase in size (244, 222, and 426 nm in liposome-coated IFX (IFX-L), Aminoclay-liposome-coated IFX (AC-IFX-L), and Eudragit S100-aminoclay-liposome-coated IFX (EAC-IFX-L), respectively) and zeta potential values were reversed (− 54.8, + 2.6, and − 31.5 mV in IFX-L, AC-IFX-L, and EAC-BSA-L, respectively) (Fig. 1b). Encapsulation efficiency were 28.9 ± 2.4% (IFX-L), 14.8 ± 0.7% (AC-IFX-L), and 6.9 ± 0.6% (EAC-IFX-L). Similar to the decrease in the concentration of nanocomposites with BSA, the entrapped concentrations of nanocomposites also decreased (2,888 ± 236, 1,480 ± 71, and 686 ± 63 μg/mL in IFX-L, AC-IFX-L, and EAC-IFX-L, respectively). Compared with BSA, IFX had a higher loading concentration, which may be due to the difference between hydrophilic and hydrophobic aa domains. IFX contains more hydrophobic parts that are more compatible with phospholipid membranes than BSA, resulting in their entrapment at higher concentrations. Collectively, these observed structural properties confirmed the successful formation of the antibody drug delivery nanocomposites of liposomes, aminoclay, and Eudragit S100.

We next assessed the physical stability of BSA-entrapping nanocomposite carriers under simulated gastrointestinal conditions by using the dialysis. The protein integrity profile of free BSA before and after exposure to simulated gastric fluid (SGF; 2 g NaCl/1L water, pH 1.2, with or without 3.2 g/L pepsin) or simulated intestinal fluid (SIF; 6.8 g KH_2_PO_4_/1 L water, pH 6.8, with or without 10 g/L pancreatin) is shown in Additional file 1: Fig. S1. When free BSA solution was incubated in SGF without pepsin supplementation, no significant change in the BSA-corresponding protein band was observed, indicating the high protein stability of BSA under the experimental conditions (Additional file 1: Fig. S1a). The complete loss of the BSA band was observed after 1-min incubation in SGF supplemented with pepsin, indicating a rapid protein degradation induced by enzymatic action (Additional file 1: Fig. S1b). In SIF supplemented with pancreatin, protein degradation was slower than control, but occurred in a time-dependent manner (Additional file 1: Fig. S1c). The decrease in the protein band of the BSA obtained from nanocomposite carriers (BSA-L, AC-BSA-L, and EAC-BSA-L) was much less and slower in both SGF and SIF (supplemented with pepsin or pancreatin), compared to free BSA solution (Fig. S1d, e).

Particle size changes and protein release profiles from the three nanocomposite carriers were also examined in PBS, SGF, or SIF (Fig. 1c). Eudragit S-100 is a pH-sensitive formulation that decomposes above pH 7, and because Eudragit-coating does not peel off in PBS and SIF, the size of the nanocomposite carriers was unchanged. All of the tested nano-formulations were observed to have delayed and reduced drug release (Fig. 1d). In particular, EAC-BSA-L showed minimal drug release in SIF for 2 h, suggesting its superior ability to retard premature release of the protein drug. EAC-BSA- L increased in size as soon as it was added to SGF. However, because there was no change in size until 8 h and there was no change of protein structure in circular dichroism data, EAC-BSA-L exists as a weak aggregate between particles under acidic conditions, but there was no change in the structure of the nanocomposite carriers. In PBS incubation, the spectra of free BSA and the other three nanocomposite-encapsulated BSAs were different (Fig. 1e). This may be due to the separation of the liposomes by adding SDS just before the circular dichroism measurement to isolate the BSA inside the liposome. Collectively, the α-helix structure of free BSA was broken under SGF (pepsin +) for 1 h. Whereas the structure of BSA with nanocomposite carriers was much better maintained than free BSA group, especially to BSA encapsulated in EAC-BSA-L remained nearly intact. These results indicate the successful fabrication of drug delivery nanocomposite carriers with the ability to safely and slowly release encapsulated cargo under gastric and intestinal conditions.

### Targeting ability of nanocomposite carriers

**Fig. 2:**
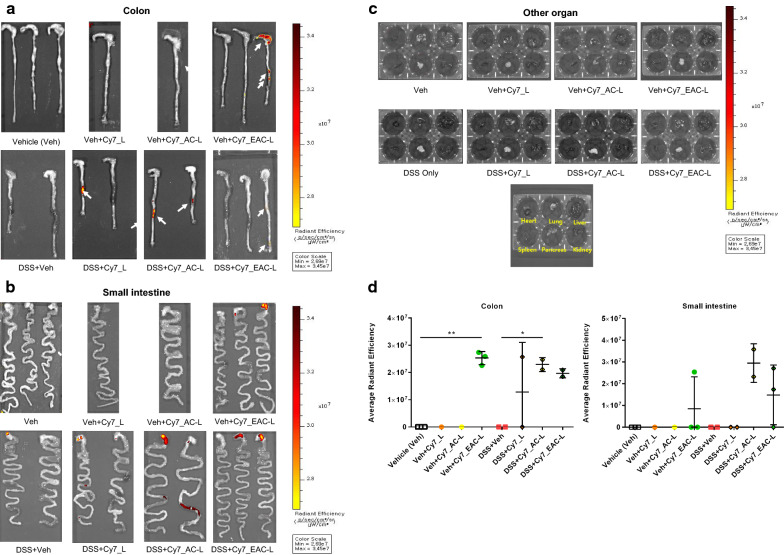
Drug delivery formulations in vivo are effectively delivered to inflamed colon and do not have a systemic effect. Images were obtained 7 h after oral administration of Cy7-labeled delivery carriers using an in vivo imaging system (IVIS). **a** Fluorescence of Cy7 measured in the inflamed colon was observed in all drug delivery carrier groups. Arrows indicate strong fluorescence signals at the site of inflammation. **b** In inflamed small intestine, the AC-L group and EAC-L group were measured for fluorescence of Cy7. **c** Fluorescence images of Cy7 in a multi-well format obtained from organs (heart, lung, liver, spleen, pancreas, and kidney). **d** Average radiant efficiency of drug delivery formulations distributed in inflamed intestine in comparison to normal conditions. Data representative of eight independent groups with *n* = 3 mice/group (Vehicle group), *n* = 1 mice/group (Vehicle with Cy7 labeled L), *n* = 1 mice/group (Vehicle with Cy7 labeled AC-L), *n* = 3 mice/group (Vehicle with Cy7 labeled EAC-L), *n* = 2 mice/group (DSS colitis with vehicle), *n* = 2 mice/group (DSS colitis with Cy7 labeled L), *n* = 2 mice/group (DSS coltis with Cy7 labeled AC-L), *n* = 2 mice/group (DSS colitis with Cy7 labeled EAC-L. Data are expressed as mean ± SD. *p < 0.05; **p < 0.01; ***p < 0.001; ****p < 0.0001. Statistical significance was assessed using Student’s t-test (**d**) and one-way ANOVA followed by Dunnett post-test. Control, treated with vehicle; DSS+Cy7_AC-L, DSS colitis with aminoclay-liposome-coated Cy7; DSS+Cy7_EAC-L, DSS colitis with Eudragit S100-liposome-coated Cy7; DSS+Cy7_L, DSS colitis with liposome-coated Cy7

To confirm targeting ability, we performed an in vivo drug targeting study using Cy7-labeled nanocomposite carriers in mice with dextran sulfate sodium (DSS)-induced colitis fed a purified diet AIN-76A (Table S1). DSS colitis model was used for simplicity and many similarities to IBD, especially human ulcerative colitis [22]. At 7 h post oral administration of the Cy7 labeled delivery carriers, fluorescence imaging showed stronger Cy7 fluorescence signal in the colon and a significant but weaker Cy7 fluorescence signal in the small intestine (Fig. 2a, b). In the cecum and colon without colitis, EAC-L group exhibited higher fluorescence signals. In contrast, other organs (heart, lung, liver, spleen, pancreas, and kidney) did not show significant Cy7 fluorescence signals (Fig. 2c). The average radiant efficiency of all nanocomposite carriers in the inflamed colon was increased. The AC-L treated mice with the inflamed colon had higher values than DSS only group and mice treated with EAC-L without colitis had higher values than control groups (Fig. 2d). These results of the biodistribution study using Cy-7 indicate no significant systemic exposure of nanocomposite carriers when orally administered to target the inflamed colon.

### Nanocomposite carriers enhance the anti-inflammatory capacity of intestinal macrophages

Peripheral blood monocytes and lymphocyte can migrate and infiltrate to the inflammatory gastrointestinal mucosa [23, 24]. The potent anti-inflammatory capacity of T cells and monocytes have a crucial function in colitis. Dysregulation of CD4 T helper cells and their signature cytokines can contribute to IBD pathogenesis [25]. TNF-α secretion is significantly increased by inflamed lamina propria monocytes in IBD patients, and the clinical severity of IBD and serum TNF-α level are correlated [26, 27]. To evaluate the effects of nanocomposite carriers (L-FITC, AC-L-FITC, and EAC-L-FITC) on lymphocytes (CD3^+^CD4^+^ cells) and monocytes (CD11b^+^ cells) in PBMCs of IBD patients, we observed a subset of CD3^+^CD4^+^ T cells and CD11b^+^ monocytes (Additional file 1: Fig. S2a). The mean fluorescence intensity (MFI) values of nanocomposite carriers in lymphocytes were between 1000 and 3000, and those of nanocomposite carriers in monocytes were between 3000 and 15,000. Lymphocytes of patients with ulcerative colitis had significantly higher MFI values in the all nanocomposite carrier groups compared to the BSA-FITC group, whereas monocytes in ulcerative colitis patients did not differ significantly from those of BSA-FITC group (Additional file 1: Fig. S2b). The EAC-L-FITC group had significantly higher MFI values in the lymphocytes and monocytes of patients with Crohn’s disease compared to the BSA-FITC group. AC-L-FITC group had significantly a higher MFI value in lymphocytes of Crohn’s disease patients than BSA-FITC group, but there was no difference in monocytes. These results suggest that nanocomposite carriers may have a higher absorption into monocytes than lymphocytes, and that absorption into lymphocytes show a significant difference between carriers.

### IFX-L, AC-IFX-L, and EAC-IFX-L delivered IFX to targeted inflamed colon and resulted in therapeutic effects

We examined the therapeutic effects of L, AC-L, EAC-L, PO-IFX, and IP-IFX (10 mg/kg) in mice with DSS-induced colitis (Additional file 1: Fig. S3a). Our results showed that liposome-treated mice had a significantly less colitis-induced body weight loss and colon shortening (Additional file 1: Fig. S3b, c). In addition, mice treated via intraperitoneal injection of IFX had a significant reduction of colon shortening (Additional file 1: Fig. S3c). These results were further supported by DAI findings (Additional file 1: Fig. S3d). In the PAS-stained colon tissues, mice treated with liposomes (L group) or IFX via intraperitoneal injection (IP-IFX group) had less colon tissue damage and inflammatory cell infiltration than those treated with other nanocomposite carriers or control group (Additional file 1: Fig. S3e). Histomorphological scores were significantly lower in in L and IP-IFX groups compared to the DSS inflammation group, whereas goblet cell scores were not significantly different between the treatment groups. These results might suggest that drug delivery via liposome encapsulation is superior to other nanocomposite carriers in terms of its anti-inflammatory effects.

**Fig. 3:**
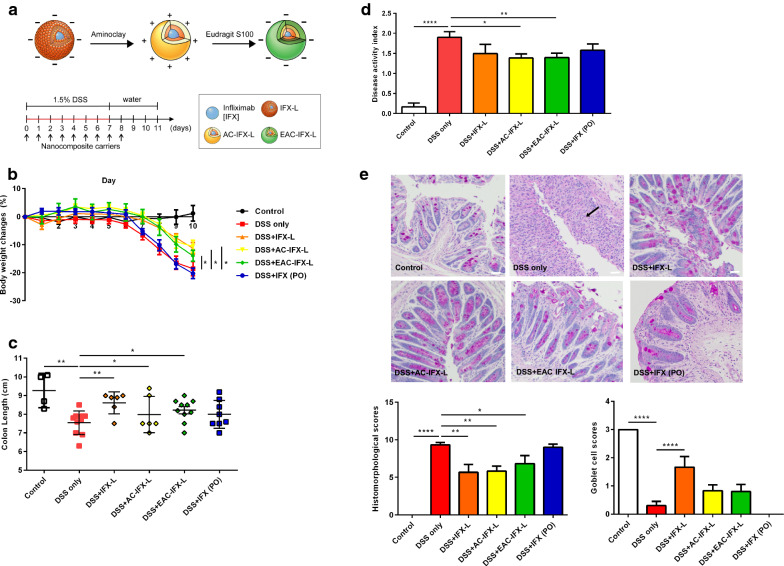
Therapeutic effects of drug delivery formulations in mice with dextran sulfate sodium-induced colitis. **a** The methods for DSS-induced colitis and oral delivery carriers with infliximab (IFX) administration, control (n = 4), DSS only (n = 10), IFX-L group (n = 6), AC-IFX-L (n = 6), EAC-IFX-L (n = 10), PO-IFX (n = 8). **b** Body weight changes of each group in DSS-induced colitis mouse model. **c** Colon length; values are represented as length (cm). **d** The clinical activity scores in the control group and treatment groups measured using the disease activity index (DAI). **e** Histopathologic features of nanocomposite carriers and IFX delivery formulations on the DSS-induced colitis mouse model, via PAS staining. Arrows indicate inflammatory cells in the lamina propria. Scale bar: 20 μm. Histomorphological scores and Goblet cell scores. Data are expressed as mean ± SD. *p < 0.05; **p < 0.01; ***p < 0.001; ****p < 0.0001. Statistical significance was assessed using Student’s t-test (**d**) and one-way ANOVA followed by Dunnett post-test

To test the therapeutic effects of nanocomposite carriers loaded with IFX, mice were orally administrated PBS, IFX-L, AC-IFX-L, EAC-IFX-L, or IFX daily (10 mg/kg, PO) from day 0 to day 8 (total 9 days). Colitis was induced by administering 1.5% DSS through drinking water for 7 days (Fig. 3a). IFX-L, AC-IFX-L, and EAC-IFX-L groups resulted in significantly less weight change, compared to the DSS-treated group (Fig. 3b). All IFX delivery formulations with nanocomposite carriers had a significantly greater therapeutic effect in terms of colon length reduction and histopathological inflammatory cell infiltration, compared to the DSS-treated group (Fig. 3c, e). There was no significant difference in colon shortening between the nanocomposite carriers themselves and the IFX encapsulated by nanocomposite carriers. There was a significantly higher improvement of DAI in the AC-IFX-L and EAC-IFX-L groups, compared to the DSS-treated group (Fig. 3d). Interestingly, EAC-IFX-L showed lower DAI value than EAC-L. All IFX delivery formulations with nanocomposite carriers showed significantly lower histomorphological scores than the DSS-treated group. The IFX-L group showed a higher goblet cell score than the DSS-treated group. AC-IFX-L group showed lower histomorphological scores and higher goblet cell scores than AC-L group. These results suggest that the conjugation of IFX to nanocomposite carriers preserves IFX function which successfully targets the colitis site.

### Anti-inflammatory effects of nanocomposite carriers loaded with IFX

**Fig. 4:**
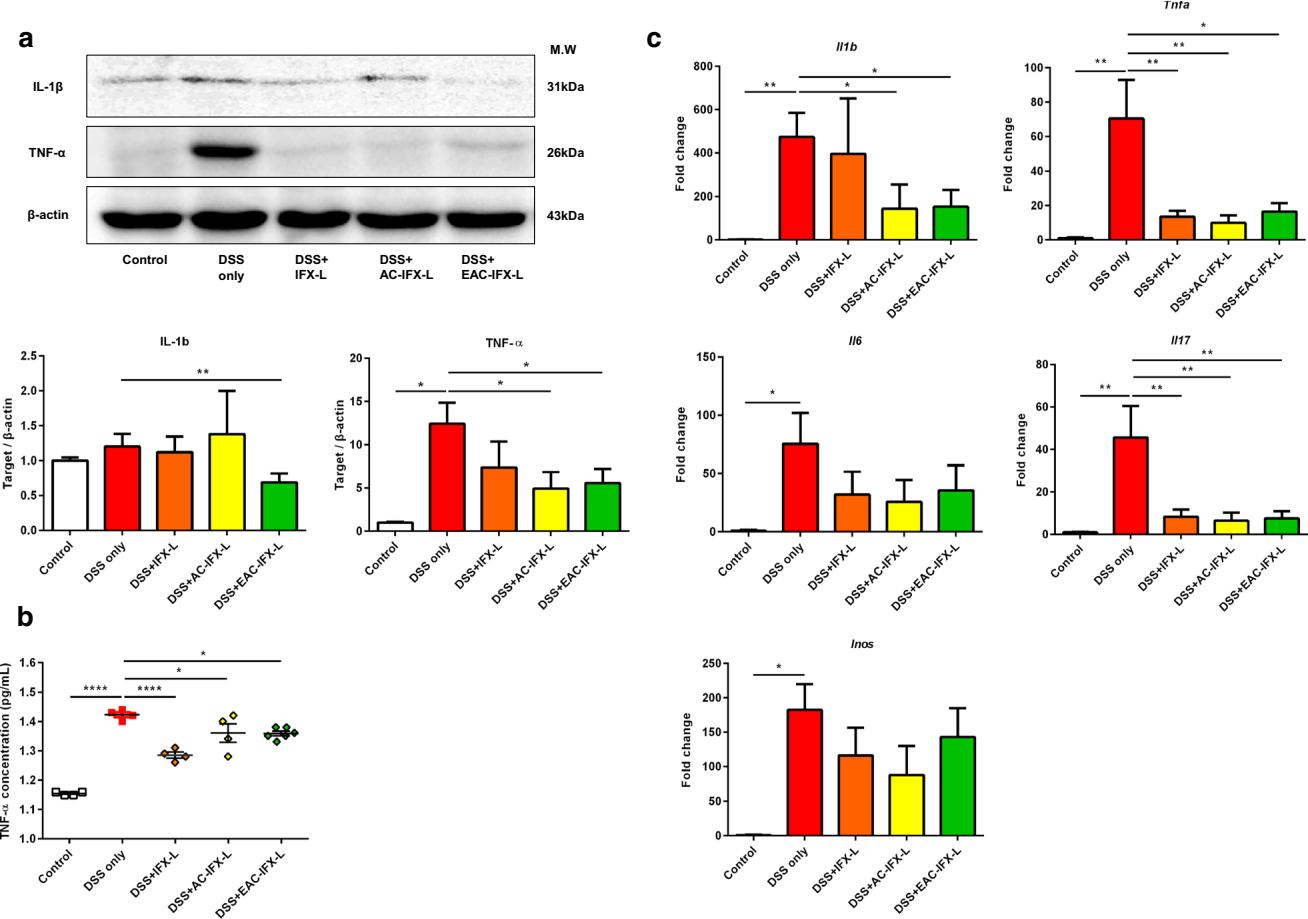
Anti-inflammatory effects of nanocomposite carriers loaded with IFX. **a** Western blot analysis of protein expression of TNF-α and IL-1β in drug delivery formulations with IFX and their effects on the production and regulation of inflammation-related factors. The ratios to beta-actin to standardize cytokine protein expression in mice with DSS-induced colitis compared to control mice. **b** Expression of TNF-α in plasma of mice with DSS-induced colitis using ELISA. **c** Expression of inflammation-related cytokines mRNA levels in the colon of mice with colitis treated by IFX using drug delivery formulations. Gene expression were evaluated by quantitative RT-PCR and relative expression was reported as fold change compared to the control by normalizing transcription level to β-actin. Data are expressed as mean ± SD. *p < 0.05; **p < 0.01; ***p < 0.001; ****p <0.0001. Statistical significance was assessed using Student’s t-test (C-I) and one-way ANOVA followed by Dunnett post-test. AC-IFX-L, aminoclay-liposome-coated IFX; EAC-IFX-L, Eudragit S100-aminoclay-liposome-coated IFX; IFX, Infliximab; IFX-L, liposome-coated IFX; control, treated with vehicle

We used western blotting to investigate the effects of IFX delivery formulations on the protein expression of TNF-α and IL-1β. Induction of inflammation by DSS increased the expression of TNF-α and IL-1β, whereas protein expression in the EAC-IFX-L group was significantly decreased (Fig. 4a). The expression of TNF-α in the AC-IFX-L group was also significantly decreased, compared to the DSS only group.

As an indicator of therapeutic effect of the IFX delivery formulation against inflammation, TNF-α expression in serum extracted from mice was measured by ELISA. In the all IFX delivery formulations with nanocomposite carrier groups, the expression levels of TNF-α were significantly decreased compared to the DSS-treated control group (Fig. 4b).

Inflammation-related factors (*Il1b*, *Tnfa*, *Il6*, *Il17,* and *Inos*) were analyzed at the mRNA level in the DSS colitis colon after receiving IFX delivery formulation (Fig. 4c). The IFX-L group exhibited significantly lower level of *Tnfa* and *Il17* compared to the DSS-treated control group. Interestingly, the levels of *Il1b* and *Il17, as well as Tnfa,* were reduced in the AC-IFX-L and EAC-IFX-L groups. All drug delivery formulations showed no significant decrease in *Inos* and *Il6* compared to the DSS-treated group. However, there was a larger fold change in mRNA levels of inflammation-related factors in the direct oral administration of IFX group without carriers than via drug delivery formulations, but this was no statistically significant difference (Additional file 1: Fig. S4). Collectively, these results suggest that the three drug delivery formulations with entrapped IFX successfully targeted *Tnfa* in the inflamed colon and had an anti-inflammatory effect in a DSS-induced colitis model.

## Discussion

Colon-targeted oral drug delivery systems are quite an attractive therapeutic strategy for the treatment of diseases affecting the colon, such as IBD [28, 29]. An ideal colon-specific delivery system maintains the maximum target-specific concentration of an entrapped drug, while minimizing systemic exposure by preventing premature drug release. The colon, a suitable delivery target site for proteinaceous drugs, has relatively limited proteolytic activity compared to other parts of the gastrointestinal tract. The residence time of drugs in the colon is also relatively longer than that of other sites [30, 31]. In this regard, the properties of surface coating materials and their interactions with liposomes are important determinants of the effects of liposome drug carriers targeting the colon. The efficiency of orally administered drugs delivered to the colon is improved by the polymer coating liposome formulation via pH-dependent release and mucosa-adhesive properties. In addition, phosphatidylcholine, a major component of the gastrointestinal membrane and liposome, reduces the inflammatory response and induces remission in patients with ulcerative colitis [32]. Aminoclay is an excellent material for improving the bioavailability of poorly water-soluble drugs. The drug-clay complexes can regulate drug release properties by interacting with negatively charged drug molecules in water. Indeed, various delivery systems have been developed using Eudragit-coated liposome or aminoclay as efficient and versatile biocompatible carriers [33, 34]. The charges of AC-L are made positive by the amino groups (-NH_3_) of aminoclay and the charges of the anionic polymer nanoparticles of EAC-L are negative because of the carbonyl groups on the polymeric chain extremities. Overall, the results of the current study showed that nanocomposite carriers can serve as efficient oral delivery carriers in the treatment of IBD.

In this study, the degradation of nanocomposites entrapped with BSA was not significant in SGF and SIF, and the release rate of BSA in PBS and SIF was lower than that of free BSA. Normal pH ranges from 5.9 in the proximal colon to 6.1 in the distal colon, However, colon pH values in IBD patients vary significantly from pH 4.8 to 7.3 [35, 36]. Because bile salts and digestive enzymes (pancreatin) have a synergistic effect on the breakdown of liposome membranes in SIF, drug release rates increase in SIF compared to SGF [28, 37]. However, in this study, the difference in diameter change between SIF and SGF was minimal. Liposomes are less susceptible to pepsin alone and stable to pancreatin at neutral pH without bile salts [38]. The reason that results of the current study differ from previous studies may be because we did not mix bile salts with digestive enzymes that could break the integrity of liposome by solubilizing the membrane and forming temporary pores. However, the current study showed that the nanocomposite carriers are stable even at different pH changes, such as in the stomach or small intestine, and can reach the colon by maintaining stable drug concentrations for a long time. The stability of BSA was analyzed by circular dichroism spectroscopy which indicated that the α-helix structure of free BSA was broken after 1 h under SGF (pepsin +). However, the BSA in nanocomposite carriers retained a much better structure than free BSA, especially in EAC-L was included.

IBD patients are characterized by altered expression of tight junction proteins and loss of barrier integrity [39, 40]. In addition, TNF-α, a pathological cytokine, increases epithelial permeability through changes in tight junction function and structure. Since IFX is administered intravenously as a monoclonal antibody, TNF is systemically antagonistic.

This method of administration is associated with several side effects and leads to the formation of antibodies to infliximab associated with loss of response to IFX and infusion responses [9–11, 41]. Daily oral treatment with IFX minimizes the decrease of TNF concentration that occurs after intravenous administration, and can continuously expose the relatively high concentrations of IFX to the confined targeted area of treatment [42, 43]. In this study, we orally administered IFX targeting the colon using liposomes, aminoclay, and Eudragit S100-based nanocomposite carriers. In previous studies, in vivo image analysis by confocal microscopy confirmed the effective intracellular distribution of fluorescently labeled nanocomposite carriers in the colon [44, 45]. The expression of each macrophage marker gene and the accumulation of liposomes showed a significant positive correlation, and liposomes enhanced the accumulation of drug candidates through macrophages in damaged colon tissues [46]. In addition, the fluorescence intensity of fluorescence-labeled liposomes is closely related to the expression levels of the macrophage marker. This indicates that the absorption mechanism of the liposomes is related to the phagocytic property of macrophages [46]. Furthermore, a previous study of nanocomposite carriers using aminoclay and Eudragit in LPS-stimulated RAW 264.7 cells, found no toxicity to macrophages [45]. The results of these publications, taken together with those of the current study, indicate that these nanocomposites may be good candidates as carriers for oral drug delivery.

Since Eudragit degrades below pH 6.8 and the gastrointestinal tract in mice is shorter than that of humans, the therapeutic effects of EAC-L and EAC-IFX-L require further study. Importantly, our work provides additional insight into the development of orally administered nanocomposite carriers for the treatment of colitis. In addition, our method of coating IFX has already proven to be an effective approach in treating IBD and has the potential to be used in personalized medicine to provide a tailored oral delivery carrier because it reaches the colon and targets macrophages with unique biocompatibility and biodegradability.

The increased secretion of TNF-α and IL1β in the colonic lamina propria has an important function in the development of IBD. Patients with ulcerative colitis have higher baseline levels of IL1β and TNF-α than in patients with Crohn’s disease [47]. IFX treatment increases the apoptosis of T lymphocytes in the intestinal mucosa [48]. The expression of *Il17* is increased in the intestinal mucosa and serum of IBD patients [49]. The reduction of *Il17* expression in the intestinal mucosa of IBD patients is closely associated with endoscopic response and mucosal healing after IFX treatment [50]. Our results indicate that drug delivery formulations significantly increased MFI values for lymphocytes in patients with ulcerative colitis, while the mRNA expression of *Tnfa*, *Il1b*, and *Il17* was significantly decreased. *Il1b* and *Il17*, which are involved in the pathogenesis of IBD due to innate lymphoid cells and CD4^+^ Th17 cells, were significantly decreased in the EAC-IFX-L group [51, 52]. Therefore, although our EAC-L data suggest that it is selectively taken by macrophages to improve DSS-induced mouse colitis, further studies on the molecular mechanisms regulating macrophage function are necessary. Our results indicate that carriers coated with liposomes alone and aminoclay-coated liposomes also contributed to anti-inflammatory responses. Further studies on the regulation of inflammatory cytokines by the carriers themselves are required to confirm their anti-inflammatory effects and those of liposomes.

## Conclusion

All three nanocomposite carriers loaded with IFX were prepared with narrow size distribution and high encapsulation efficiency. Theses nanocomposite carriers loaded with IFX provide a colon-specific anti-inflammatory effect without adversely affecting systemic homeostasis. Oral drug delivery by EAC-L may be a novel means of transporting large molecule drugs to the intestines in a non-cytotoxic way, to stimulate CD4^+^ T cells as well as macrophages. Furthermore, the unique propensity of EAC-L enhances anti-inflammatory responses of intestinal macrophages and provides attractive strategies for preventing or treating autoimmune diseases. All the three nanocomposite carriers loaded with IFX had better colitis improvement than the control group. In addition, IFX treatment by AC-IFX-L and EAC-IFX-L-based nanocomposites not only showed a significant anti-inflammatory effect, but also remarkably decreased TNF-α level in a DSS-induced mouse colitis model. This suggests that these oral anti-TNF delivery systems be a promising therapeutic approach for treating IBD.

## Methods

### Reagents and materials

1,2-Dimyristoyl-sn-glycero-3-phosphocholine (DMPC) and 1,2-dimyristoyl-sn-glycero-3-phosphorylglycerol sodium salt (DMPG) were purchased from Avanti Polar Lipid Inc. (Alabaster, AL, USA). Cholesterol (Chol), α-tocopherol, bovine serum albumin (BSA) fluorescein isothiocyanate conjugate (FITC-BSA), ammonium persulfate, pepsin, pancreatin, and Remicade^®^ (IFX) were purchased from Sigma-Aldrich (St. Louis, MO, USA). Cy™7 Mono NHS Ester was purchased from GE Healthcare (Little Chalfont, Buckinghamshire, UK). BCA Protein Assay Kit and PageRuler Prestained Protein Ladders were purchased from Thermo Fisher Scientific (Waltham, MA, USA). Sodium dodecyl sulfate (SDS) was purchased from Kracker Scientific (NY, USA). Coomassie-brilliant blue R-250 staining solution, 30% acrylamide/Bis solution (29:1), TEMED, 4 × Laemmli sample buffer, 1.5 M Tris–HCl buffer and 0.5 M Tris–HCl buffer were purchased from Bio-Rad Laboratories (Hercules, CA, USA.). Magnesium chloride hexahydrate (98%) and other inorganic salts were purchased from Junsei Chemical Co. (Tokyo, Japan). Eudragit^®^ S100 was kindly donated by Evonik Korea (Seoul, Korea).

### Preparation of liposomes

DMPC, Chol, DMPG, and α-tocopherol were mixed at a molar ratio of 26:10:2:2 in tertiary butyl alcohol. The mixtures were frozen at − 80 °C overnight followed by freeze-drying (EYELA FDU-1200, Tokyo, Japan) for 24 h. The lipid cakes (total 40 μmol of lipid mixture) obtained were hydrated with 1 mL of IFX solution, which was obtained by dissolving Remicade powder in distilled water (10 mg/mL), or FITC-BSA solution in phosphate-buffered saline (pH 7.4). The hydrated liposome dispersions were vortexed and sonicated for 1 h at 24 °C of DMPC by using an ultrasonic cleaning bath (Branson^®^, 3510-DTH Ultrasonic Cleaner, Danbury, CT, USA). To obtain a liposomal dispersion with increased homogeneity and reduced particle size, additional sonication was carried out by using a cell disruptor (Bioruptor^®^, UCD-200T, Cosmo Bio Co., Tokyo, Japan) as previously described [53]. Liposome dispersions were then subjected to freeze-thawing for five cycles of 5 min incubation at − 180 °C and 15 min incubation at 37 °C to improve the protein encapsulation. To remove non-encapsulated proteins, liposomes were collected by centrifugation at 43,000×*g* for 1 h at 4 °C. The resulting pellets were re-suspended in the original volume of phosphate-buffered saline (PBS). The prepared liposomes were stored at 4 °C until use.

### Coating of liposomes

The 3-aminopropyl-functionalized magnesium phyllosilicate (aminoclay) was synthesized by following a method described previously [45]. Before coating, the bulk aminoclay powder was dispersed in water, followed by ultrasonication for 10 min, for the exfoliation of the aminoclay. Aminoclay-coated liposomes (AC-L) were obtained by spontaneous assembly of positively charged aminoclay on negatively charged liposomal surfaces. Briefly, equal volumes of liposome dispersions pre-diluted with distilled water to give a lipid concentration at 10 mg/mL were added drop-wise to exfoliated aminoclay dispersion (10 mg/mL) to give a lipid/clay weight ratio of 1:1. The mixture was incubated at 25 °C for 30 min with stirring and then centrifuged at 15,000×*g* for 7 min at 4 °C. The resulting aminoclay-liposome pellets were re-suspended in 1 mL of PBS.

For further coating of clay-liposomes with Eudragit S-100, an equal volume of clay-liposome dispersions pre-diluted to a concentration of 2.5 mg lipid per mL was added drop-wise to 0.1% Eudragit S100 solution in PBS. The mixture was incubated at 4 °C for 30 min with stirring and then centrifuged at 15,000×*g* for 7 min at 4 °C. The resultant Eudragit S100-coated aminoclay-liposome (EAC-L) pellets were re-dispersed in the original volume of PBS.

### Physicochemical characterization of liposomes

The mean particle size and polydispersity index of nanocomposite carriers were measured by dynamic light scattering using a fiber-optics particle analyzer (FPAR-1000, Otsuka Electronics, Osaka, Japan) as described in our earlier studies [15]. Particle size analysis data were assessed using the CONTIN program provided by the manufacturer. Zeta potential (the electrical potential at the shear plane of the nanoparticle) was measured using a Zetasizer Nano ZSP (Malvern, UK). Samples were diluted 50-fold with deionized water before measuring to reach the analytical measurement range. Default instrument settings and automatic analysis were used for all measurements. Each measurement was carried out in duplicate.

The diameter and morphology of liposomes were imaged by negative-stain transmission electron microscopy. Liposome samples were 50-fold diluted with PBS solution and dropped on a 200-mesh copper grid coated with carbon and negatively stained with 2% uranyl acetate for 1 min. Excess stain was removed and the samples were allowed to air-dry completely. Dried samples were examined using a Tecnai G2 Spirit (FEI Company, Hillsboro, OR, USA) operating at 120 keV.

The encapsulated concentration of IFX and FITC-BSA were determined by BCA protein assay or by measuring the fluorescence of FITC (excitation 490 nm, emission 525 nm) with a fluorescence spectrometer (FS-2, Scinco Ltd., Seoul, Korea) after disrupting the liposome dispersions with an equal volume of 10% SDS (fluorescence assay) or ethanol (BCA assay). Standard curves pre-constructed with serial dilutions of FITC-BSA with ethanol were used to convert fluorescence to FITC-BSA concentration. The stability of BSA in the nanocomposite carriers was investigated using circular dichroism spectroscopy. Gastrointestinal stability of liposomes and protein stability analysis are described in the Supplementary Information.

### Animal studies

#### Experimental animals

Eight-week-old C57BL/6 mice were kept under standard conditions at 21–22 °C under 12 h light/dark cycle and allowed to acclimate for a week before starting the experiment. Body weight and physical activity were monitored daily.

### Distribution of nanocomposite carriers in dextran sulfate sodium colitis mice

To assess the biodistribution of nanocomposite carriers after oral administration, Cyanine-7 (Cy7)-labeled nanocomposite carriers were prepared. Briefly, two μg of Cy7 was dissolved with 40 μmol of DMPC:Chol:DMPG:α-tocopherol (26:10:2:2) mixture in tertiary butyl alcohol. The liposomes were prepared from the mixture as described above, except that the un-entrapped Cy7 was separated from the liposomes by dialysis. Each liposome dispersion was adjusted to 0.44 μg/mL before oral administration.

Colitis was induced in mice by oral administration of 1.5% (wt/vol) DSS (36-50 KD molecular weight, MP Biomedicals, Solon, OH, USA) for 5 days in drinking water [54]. After fasting for 12 h, Cy7.0-L, Cy7-AC-L, and Cy7-EAC-L were orally administered to mice with DSS-induced colitis at a dose of 20 mg/kg in a volume of 100 μL PBS. Control mice maintained normal drinking water for 5 days and then Cy7.0-EAC-L was administrated orally at the same dose. Mice were sacrificed 7 h after administration of Cy7.0-labeled nanocomposite carriers. Cy7.0-labeled nanocomposite carriers were visualized (Cy7: excitation 750 nm, emission 773 nm) and analyzed by using an in vivo image analyzer (Caliper IVIS Lumina II, Caliper Life Science, USA).

### DSS-induced colitis and therapeutic effect of nanocomposite carriers

To test the therapeutic effect of nanocomposite carriers, colitis was induced in mice by administering 1.5% DSS in their drinking water for 7 days [22, 55]. Mice were randomly divided into five different groups: DSS only (DSS-treated control group), DSS-treated with L (L group), DSS-treated with AC-L (AC-L group), DSS-treated with EAC-L (EAC-L group), DSS-treated with intraperitoneal IFX (IP-IFX group), and DSS-treated with per-oral IFX (PO-IFX group). Mice in the L, AC-L, and EAC-L groups were orally administered nanocomposite carriers for 9 days, from day 0. The dose of nanocomposite carriers (10 mg/kg for all groups) was predetermined to have an optimal therapeutic effect. Mice received 4 mg/kg of IFX in 200 μL PBS daily by oral or intraperitoneal administration for nine consecutive days. The dose of IFX was determined to have an optimal therapeutic effect based on existing studies [56, 57]. Drinking water was replaced with pure water and maintained for 2 days. Changes in body weight, stool consistency, and presence of blood in the stool or at the anus were measured daily throughout the study period. Mice were sacrificed at day 12; spleen and colons were collected to assess the therapeutic efficacy of the nanocomposite carriers. Disease activity index (DAI) was evaluated using the summed score of three factors (weight loss, stool consistency, and bleeding) [58].

### DSS-induced colitis and the therapeutic effect of nanocomposite carriers-IFX conjugates

To test the therapeutic effect of nanocomposite carriers loaded with IFX, colitis was induced in mice via the addition of 1.5% DSS to drinking water for 7 days. Drinking water was then replaced with pure water for 4 days. All mice were sacrificed on day 11. Mice were orally administered, 200 μL PBS, IFX-L (10 mg/kg), AC-IFX-L (10 mg/kg), EAC-IFX-L (10 mg/kg) or PO-IFX (10 mg/kg), once daily, from day 0 to day 8 (total 9 days). The dose of nanocomposite carriers and IFX was predetermined to have an optimal therapeutic effect [56, 57]. Flow cytometry, qRT-PCR, analysis of TNF-α by enzyme-linked immunosorbent assay (ELISA), and western blotting are described in the Supplementary Information.

### Histology

Colon tissues were fixed in 10% neutral formalin and then embedded in paraffin. Tissues were stained by hematoxylin and eosin and periodic acid Schiff (PAS) staining. The severity of colitis was scored as described previously [22]. Goblet cell staining was scored from 0 to 3 (3, minimal, < 20%; 2, mild, 21–35%; 1, moderate, 36–50%; 0, marked, > 50%).

### Goblet cell counting

Colonic tissue sections were processed with PAS staining. Stained goblet cells were counted per crypt. Maximum 52 and minimum 20 fully conserved crypts on each section were examined, and the average numbers were marked as a representative goblet cell count of each section.

### Statistical analysis

The data were reported as mean and standard deviation. Comparison among groups was done by performing Student’s *t*-tests or one-way analysis of variance (ANOVA) followed by Dunnett post-test using GraphPad Prism software (La Jolla, CA, USA). Results with a *p* value < 0.05 were reported as statistically significant.

## Supplementary information

**Additional file 1.**

## Data Availability

All data and material are included in the article and its additional files.
