# Supplementary material for: Nanocomposites-based targeted oral drug delivery systems with infliximab in a murine colitis model
Source: J Nanobiotechnology. 2020 Sep 15;18:133. doi: 10.1186/s12951-020-00693-4 (PMC7493402; doi:10.1186/s12951-020-00693-4)
Supplement: Supplementary file 1 — Additional file 1. [file 12951_2020_693_MOESM1_ESM.docx]

**Additional file 1**

**Nanocomposites-Based Targeted Oral Drug Delivery Systems with Infliximab in a Murine Colitis Model**

Jung Min Kim^1,2†^, Da Hye Kim^1,2†^, Hyo Jeong Park^3^, Hyun Woo Ma^1,2,4^, I Seul Park^1,2,4^, Mijeong Son^1,2,4^, So Youn Ro^3^, Seok Mann Hong^3,5^, Hyo Kyung Han^6^, Soo Jeong Lim^3^, Seung Won Kim^1,2,4*^, Jae Hee Cheon^1,2,4*^

^1^Department of Internal Medicine and Institute of Gastroenterology, Yonsei University College of Medicine, Seoul, Republic of Korea

^2^Brain Korea 21 PLUS Project for Medical Science, Yonsei University College of Medicine, Seoul, Korea

^3^Department of Integrated Bioscience and Biotechnology, Sejong University, 209 Neungdong‑ro, Gwangjin‑gu, Seoul, Korea

^4^Severance Biomedical Science Institute, Yonsei University College of Medicine, Seoul, Republic of Korea

^5^Department of Integrative Bioscience and Biotechnology, Institute of Anticancer Medicine Development, Sejong University, Seoul, 05006, Republic of Korea

^6^College of Pharmacy, Dongguk University-Seoul, Dongguk‑ro‑32, Ilsan‑donggu, Goyang, Korea

*Correspondence: GENIUSHEE@yuhs.ac; swk21c@hanmail.net

| **S1. Experiment methods** | **p.3** |
| --- | --- |
| **S1.1. Gastrointestinal stability of liposomes** | **p.3** |
| **S1.2. Protein stability analysis** | **p.3** |
| **S1.3. Flow cytometry** | **p.4** |
| **S1.4. Quantitative reverse-transcription polymerase chain reaction (qRT-PCR)** | **p.5** |
| **S1.5. TNF-α detection** | **p.5** |
| **S1.6. Western blotting** | **p.6** |
|  |  |
| **S2. Results** | **p.6** |
| **S2.1. Table S1 Mouse diet In Vivo Optical Imaging System (IVIS)** | **p.6** |
| **S2.2. Table S2 Patient characteristics.** | **p.7** |
| **S2.3. Table S3 Human Antibodies_ Fluorescence-activated cell sorting (FACS)** | **p.8** |
| **S2.4. Table S4 Primer sequences for qRT-PCR** | **p.8** |
| **S2.5. Table S5 Mouse Antibodies_Western-blot** | **p.8** |
| **S2.6. Fig. S1 Protein stability** | **p.9** |
| **S2.7. Fig. S2 Drug delivery formulations *in vitro*** | **p.11** |
| **S2.8 Fig. S3 Therapeutic effect of nanocomposite carriers.** | **p.12** |
| **S2.9 Fig. S4 Comparison of mRNA levels between oral administration of IFX and IFX itself** | **p.15** |

**S1. Experiment methods**

**S1.1. Gastrointestinal stability of liposomes**

To evaluate the physical stability of liposomes in the gastrointestinal tract, time-dependent protein release from bovine serum albumin (BSA)-encapsulated by liposomes in simulated gastrointestinal conditions was assessed using dialysis. Briefly, 900 μL of each liposome dispersion (BSA-L, AC-BSA-L, and EAC-BSA-L) and free FITC-BSA solution pre-adjusted to 250㎍ of BSA per mL were placed in a Float-A-Lyzer (Spectrum Labs, Rancho Dominguez, CA, USA; MWCO 1,000 kDa) and dialyzed with 30 mL PBS, simulated gastric fluid (SGF; 2 g NaCl/1 L water, pH 1.2) or simulated intestinal fluid (SIF; 6.8 g KH_2_PO_4_/1 L water, pH 6.8) at 37°C, respectively. At pre-determined time points, aliquots of each sample were taken from the media and the amount of FITC-BSA released from liposomes was quantified by measuring the fluorescence as described above. The gastrointestinal stability of liposomes was evaluated by monitoring changes in the sizes of liposomes (50 μL) incubated with 950 μL of PBS, SIF or SGF at 37°C.

**S1.2. Protein stability analysis**

To assess the gastrointestinal stability of the protein encapsulated in liposomes, after incubation of BSA-encapsulated liposomes with SIF or SGF, gel electrophoresis was performed. FITC-BSA (400 μg/mL) as a free solution or encapsulated in liposomes (liposome, Clay-liposome, and E100-Clay-liposome) was incubated with an equal volume of PBS, SGF (with or without supplementation with 3.2 g/L pepsin), and SIF (with or without supplementation with 10 g/L pancreatin) at 37°C for a designated time. Aliquots taken from each sample were mixed with 4× sample buffer, and then 20 μL of the mixture was loaded on an 8% SDS-polyacrylamide gel. Electrophoresis of samples was performed at a constant voltage of 120 volts using a PowerPac BasicTM (Bio-Rad) in SDS-PAGE buffer (25 mM Tris, 18.8 g glycine, 1 g SDS, and 1 L distilled water). After electrophoresis, the gels were stained with Coomassie-brilliant blue R-250 solution for 1 h and then destained in destaining buffer (aqueous solution of 40% methanol and 10% acetic acid). The percentage of intact parent protein (FITC-BSA) at different time intervals was calculated as follows:

Intact BSA% = D_t_/D_T_ x 100,

where Dt is the intensity of the intact FITC-BSA at time interval t and DT is the intensity of the intact FITC-BSA before digestion.

**S1.3. Flow cytometry**

Whole blood from four patients with ulcerative colitis (UC) and five with Crohn’s disease (CD) was collected in EDTA tubes and processed shortly after collection. Whole blood in EDTA (10 mL), was diluted with 10 mL of Dulbecco's PBS and loaded into Leucosep tubes (Greiner Bio-One, Frickenhausen, Germany). Human peripheral blood mononuclear cells (PBMC) were purified by Ficoll-Paque density gradient centrifugation. Cells were separated at 1,500 × g for 15 min at 4°C according to the manufacturer’s instruction. The supernatant was transferred to a new tube, centrifuged at 200 × g for 5 min at room temperature, then gently poured off and discarded. The remaining red blood cells were incubated in 10 mL of lysis buffer for 30 min at room temperature in the dark. Cells were separated at 200 × g for 5 min at room temperature, then supernatant was carefully removed. The PBMCs were washed in Dulbecco's PBS and centrifuged at 200 × g for 5 min at room temperature. Five milliliters of culture medium (RPMI 1640 containing 1% penicillin/streptomycin and 10% FBS) were diluted to 1/10 and PBMCs underwent MACS® (Miltenyi Biotec, Bergisch Gladbach, Germany) bead isolation. PBMC cultures (1×10^5^ - 1×10^6^ cell/mL) were treated with 4 types of drug delivery carriers (free BSA, BSA-L, AC-BSA-L, and EAC-BSA-L) 8 ㎍/mL (1×10^5^ cells) and incubated at 37°C for 4 h. Cell suspensions were stained on ice for 30 min in the dark with various combinations of directly fluorochrome-conjugated antibodies, CD3 (V500), CD4 (PerCP/Cy 5.5), or CD11b (APC), in permeabilization buffer (BD). Flow cytometry analysis was performed using a FACSVerse flow cytometer (BD Biosciences) and analyzed using FlowJo software (Tree Star, San Carlos, CA, USA). Patient characteristics are described in Table S2 and human antibodies to cd3, cd4, and cd11b are described in Table S3.

**S1.4. Quantitative reverse-transcription polymerase chain reaction (qRT-PCR)**

Total RNA was extracted using TRIzol Reagent (Life Technologies, Carlsbad, CA, USA) following the manufacturer’s instructions. RNA was reverse transcribed using a High Capacity cDNA Reverse-Transcription Kit (Applied Biosystems, Warrington, UK) according to the manufacturer’s protocol. qPCR was performed using SYBR Green Master Mix (Applied Biosystems, Warrington, UK) in a StepOne Plus real-time PCR system (Applied Biosystems, Warrington, UK). The thermal cycles were: 40–45 cycles of 95°C for 30 s, 60–63°C for 30 s, and 72°C for 40 s. All PCRs were run in triplicate. Relative expression was determined by the 2^–ΔΔCt^ method and results were reported as fold changes, compared to the control by normalizing transcription levels to β-actin. *Tnfa* and inducible nitric oxide synthetase (*iNos*) primers were purchased from Bioneer (AccuTarget, Daejeon, Korea). Other PCR primers are listed in Table S4.

**S1.5. TNF**-α **detection**

TNF-α was measured using a Quantikine**^®^** ELISA mouse Kit (Cat. number: MTA00B, R&D Systems, Inc., MN, USA) following the manufacturer’s instruction.

**S1.6. Western blotting**

Proteins were extracted from colon tissues using Pierce RIPA buffer (Cat# 89900, Thermo Fisher Scientific, Pittsburgh, PA, USA) mixed with Halt Protease & Phosphatase Inhibitor Cocktail (Thermo Fisher Scientific). Quantified samples were prepared using a bicinchoninic acid protein assay kit (Pierce, Rockford, IL, USA) and sample buffer with β-mercaptoethanol (Sigma–Aldrich, St. Louis, MO, USA). Prepared samples were boiled at 95°C for 5 min and centrifuged at 13,000 *g*/min for 15 min at 4°C. The prepared protein samples (20 μg) were loaded onto a polyacrylamide gel divided into stacking (5%) and running (10%) portions. Electrophoresis was performed in Tris-glycine SDS buffer (iNtRON Biotechnology, Sungnam, Gyeonggi, Korea). Differentiated proteins were transferred to polyvinylidene difluoride membranes for 90 min at 100-150 volts (Bio-Rad) and blocked with Tris-buffered saline with Tween-20 supplemented with 5% filtered-BSA. Primary antibodies (TNF-α, 1:2,000, Santa Cruz, CA, USA; IL-1β, 1:2,000, Santa Cruz; β-actin, 1:1,000, Santa Cruz) were diluted in 5% filtered-BSA and incubated with rocking overnight at 4°C. Membranes were washed and incubated with horseradish peroxidase (HRP)-conjugated secondary antibodies (TNF-α, goat anti-mouse IgG-HRP, 1:1,000; IL-1β, goat anti-rabbit IgG-HRP, 1:1,000) diluted in 5% filtered-BSA for 3 h. Anti-β-actin (Santa Cruz) was used as the loading control. Mouse antibodies are listed in Table S5.

**S2. Results**

**S2.1. Table S1 Mouse diet_** **In Vivo Optical Imaging System (IVIS)**

| Abs | Information | | |
| --- | --- | --- | --- |
| Mouse | | |  |
| AIN-76A Purified Diet | | ENVIGO | |
|  |  | Cat NO: 170481 | |

**S2.2.**

| **Table S2** **Patient characteristics.** | | |
| --- | --- | --- |
|  | CD | UC |
| *n* (male/female) | 5 (3/2) | 4 (2/2) |
| Age^a^ | 25.0 ± 1.5 | 41.3 ± 6.5 |
| Disease duration of UC / CD^a^ | 5.3 ± 2.4 | 1.5 ± 1.3 |
| UCAI/CDAI^a^ | 178.0 ± 72.5 | 3.8 ± 1.4 |
| ESR, mm/h^a^ | 19.6 ± 12.5 | 8.3 ± 2.4 |
| CRP, mg/L^a^ | 4.1 ± 2.2 | 0.8 ± 0.2 |
| Disease location^b^ |  |  |
| Ileum | 0 |  |
| Colon | 0 |  |
| Ileocolonic | 5 (100) |  |
|  |  |  |
| Cecum |  | 1 (25) |
| sigmoid colon |  | 2 (50) |
| Rectum |  | 1 (25) |
| Prior intestinal resections | 0 | 0 |
| Medication^b^ |  |  |
| Sulfasalazine/5-ASA | 2 (40) | 4 (100) |
| Immunosuppressive treatment | 1 (20) | 0 |
| Corticosteroids | 0 | 2 (50) |
| Anti-TNFα | 1 (20) | 0 |
| Anti-IL-12/IL-23 | 1 (20) | 0 |
| 5-ASA: 5-aminosalicylates; CD: Crohn's disease; CDAI: Crohn’s Disease Activity Index; CRP, c-reactive protein; ESR: erythrocyte sedimentation rate; UC: ulcerative colitis; TNF: tumor necrosis factor. ^a^Year: median ± SD ^b^*N* (%) | | |

**S2.3.**

**Table S3 Human Antibodies_** **Fluorescence-activated cell sorting (FACS)**

| Abs | Information |
| --- | --- |
| Human | |
| CD3 | BD bioscience |
|  | Clone number : UCHT1 |
| CD4 | ebioscience |
|  | Clone number : OKT4 (OKT-4) |
| CD11b | ebioscience |
|  | Clone number : ICRF44 |

**S2.4.**

**Table S4** Primer sequences for qRT-PCR

| Gene | Sequence (5′–3′) |
| --- | --- |
| Mouse | |
| *Tnfα* | F: CAAAGGGAGAGTGGTCAGGT |
|  | R: ATTGCACCTCAGGGAAGAGT |
| *Il1b* | F: GCA ACT GTT CCT GAA CTC AAC T |
|  | R: ATC TTT TGG GGT CCG TCA ACT |
| *Il6* | F: TTG CCT TCT TGG GAC TGA TG |
|  | R: CCA CGA TTT CCC AGA GAA CA |
| *Il17* | F: CAG CGA TCA TCC CTC AAA G |
|  | R: CAG GAC CAG GAT CTC TTG CTG |
| *Inos* | F: GGC AGC CTG TGA GAC CTT TG |
|  | R: GCA TTG GAA GTG AAG CGT TTC |
| *β-Actin* | F: CATCTTCACCGTTCCAGT |
|  | R: GTCCACCTTCCAGCAGAT |
| F: forward primer, R: reverse primer | |

**S2.5.**

**Table S5 Mouse Antibodies_Western-blot**

| Abs | Information | |
| --- | --- | --- |
| Mouse | |  |
| TNFα | TNFα (52B83): sc-52746 (Santa Cruz) | |
|  | Secondary: goat-anti-mouse-IgG-HRP (Santa Cruz) | |
| Il1b | Il1b (H-153): sc-7884 (SantaTA Cruz) | |
|  | Secondary: goat-anti-rabbit-IgGHRP (Santa Cruz) | |
| β-actin | β-actin (C4): sc-4778 (Santa Cruz) | |
|  | Secondary: goat-anti-mouse-IgG-HRP (Santa Cruz) | |

**S2.6.**

**a b**

**
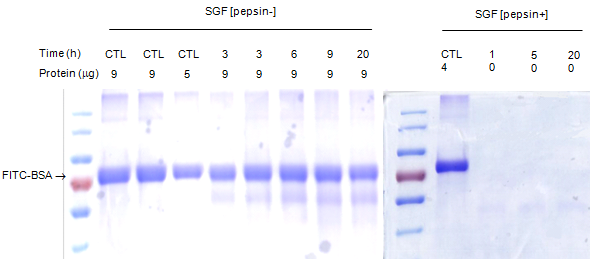
**

**c**

**
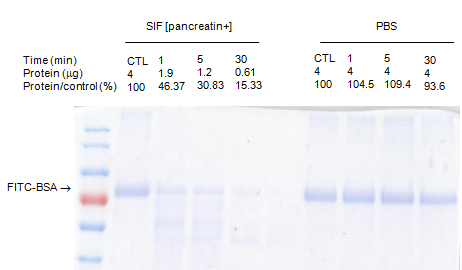
**

**d**

**
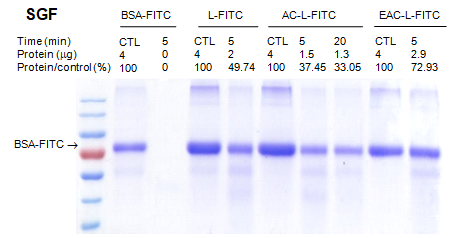
**

**e**

**
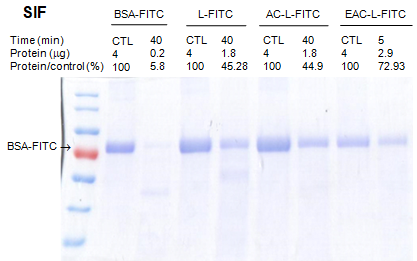
**

**Fig. S1** Protein stability of free bovine serum albumin (BSA) incubated in (A) simulated gastric fluid (SGF, without pepsin), (B) SGF (with pepsin) and (C) simulated intestinal fluid (SIF, with pancreatin) or PBS at 37°C for indicated time periods. For (B) and (C), samples corresponding to 4 μg FITC-BSA were subjected to gel electrophoreses. The molecular weight of FITC-BSA was 70 KDa. SDS-PAGE gel stained by Coomassie blue R-250. Protein stability of BSA incubated in (D) SGF (with pepsin) and (E) SIF (with pancreatin) as a free form or as a form encapsulated in liposomes (uncoated, aminoclay-coated or Eudragit S100-aminoclay-coated liposomes). Samples corresponding to 4 μg FITC-BSA were subjected to gel electrophoreses.

**S2.7.**

**a**

**
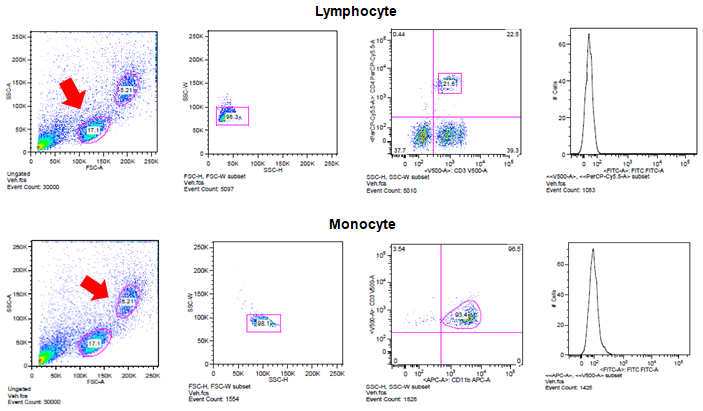
**

**b**

**
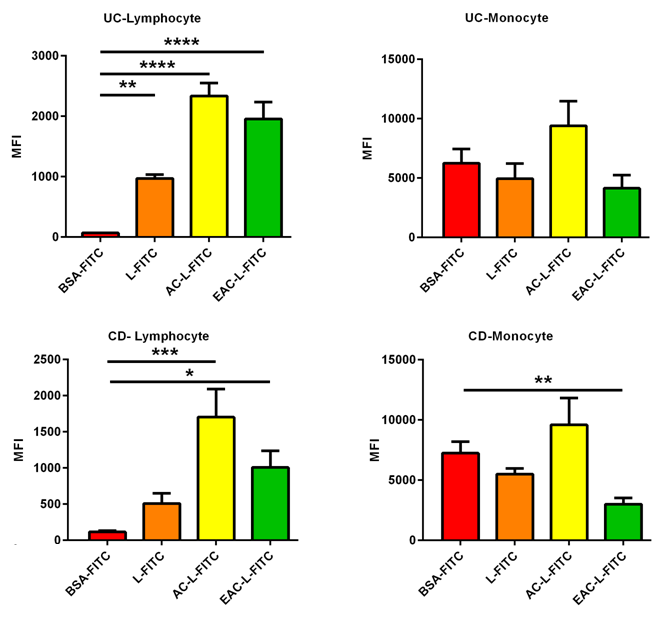
**

**Fig. S2 Drug delivery formulations *in vitro* are effectively delivered to lymphocytes and monocytes produced by the peripheral blood mononuclear cells of patients with ulcerative colitis (*n* = 4) and Crohn’s disease (*n* = 5).**

(a) Representative flow cytometry dot plot. Diagrams of flow cytometry gating the lymphocytes and monocytes of UC and CD patients. CD subset of T lymphocyte (CD3 and CD4) and macrophage (CD11b) in produced by peripheral blood mononuclear cells (PBMC) of IBD patients (b) median fluorescence intensities (MFIs) of BSA-FITC with drug delivery formulations between UC and CD patients. Data are expressed as means ± SD. *, *p* < 0.05; **, *p* < 0.01; ***, *p* < 0.001; ****, *p* < 0.0001. Statistical significance was assessed using Student’s t-test (d) and one-way ANOVA followed by Dunnett post-test. AC-L-FITC, aminoclay-liposome-coated FITC; BSA-FITC, bovine serum albumin-coated FITC; L-FITC, liposome-coated FITC; EAC-L-FITC, Eudragit S100-liposome-coated FITC

**S2.8.**

**a**

**
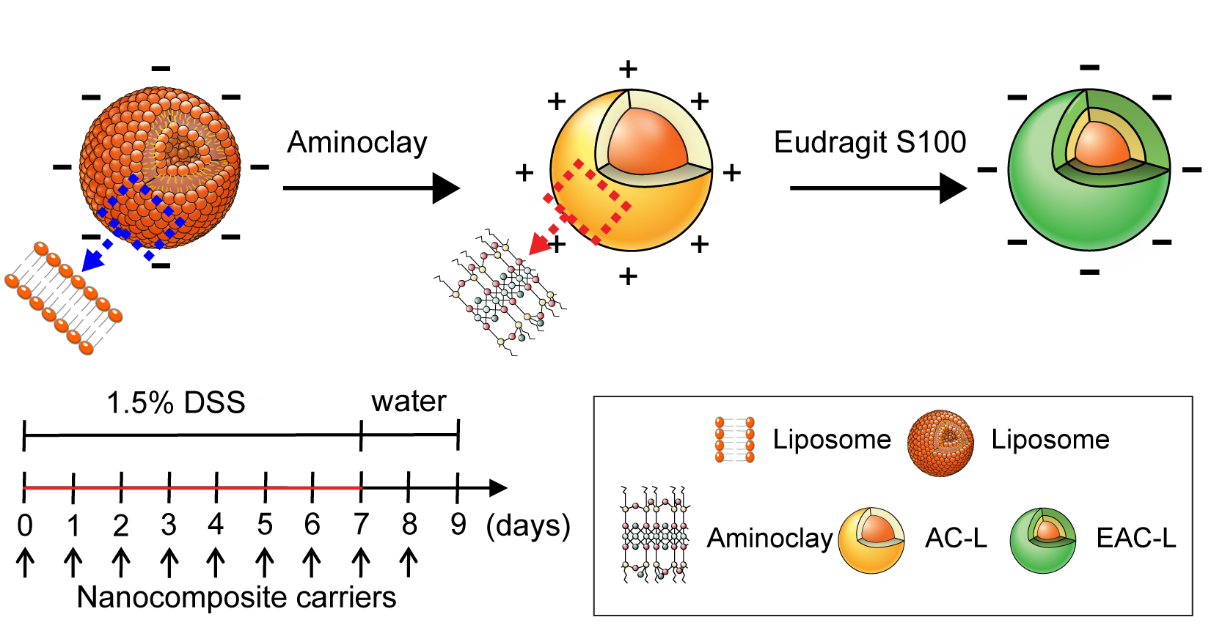
**

**b c**

**
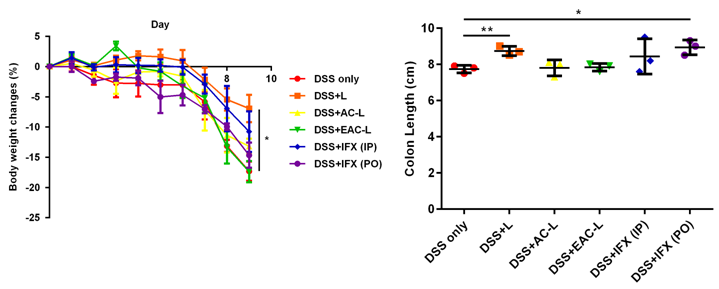
**

**d**

**
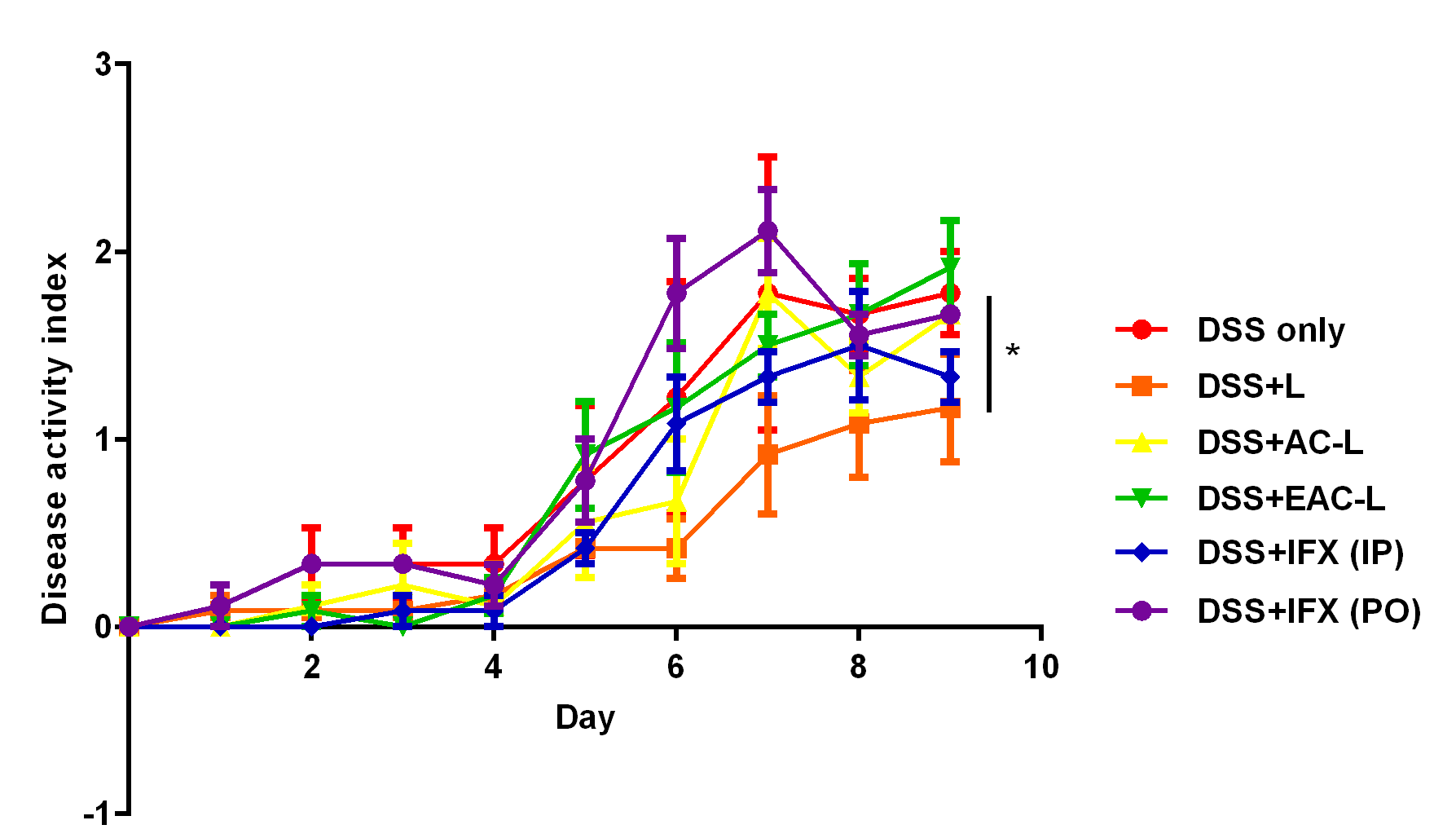
**

**e**

**
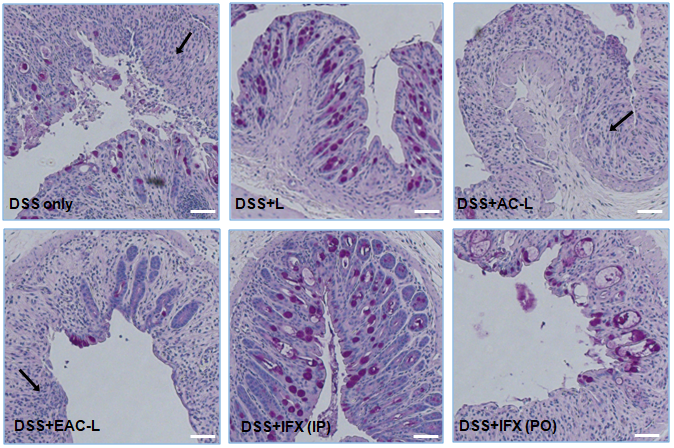
**

**
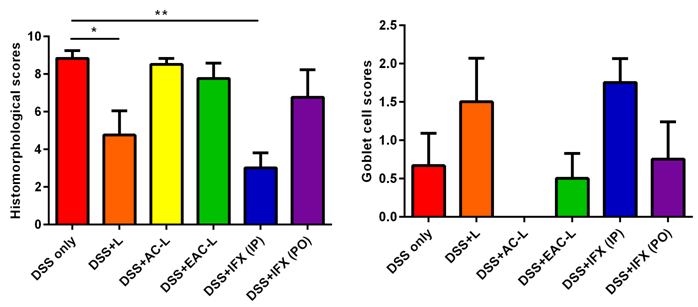
**

**Fig. S3** Therapeutic effect of nanocomposite carriers. Drug delivery system (DDS) ameliorates changes associated with inflamed mucosa in dextran sodium sulfate (DSS) colitis mice. C57/B6 mice were treated with vehicle control (*n* = 3), liposome (*n* = 4), aminoclay + liposome (*n* = 4), eudragitS100 + aminoclay + liposome (*n*=4), intraperitoneal infliximab, (*n* = 4) and oral infliximab (*n* = 4). (a) Methods of administration of DSS-induced colitis and oral delivery carriers. (b) Body weight changes of each group in a DSS colitis mice model. (c) Colon length; values are represented as length (cm). Groups of DSS with liposome and DSS treated by oral infliximab showed significantly longer colons compared to DSS only group. (d) Histopathologic features of formulations of the DSS colitis mice model, via PAS staining. Arrows indicated inflammatory cells in the lamina propria. Scale bar: 20 μm. (e) Clinical activity scores in control group and treatment groups as measured using disease activity index (DAI), histomorphological score and goblet cell score. Data are expressed as means ± SD. * *p* < 0.05; **, *p* < 0.01; ***, *p* < 0.001; ****, *p* < 0.0001.

**S2.9.**

**
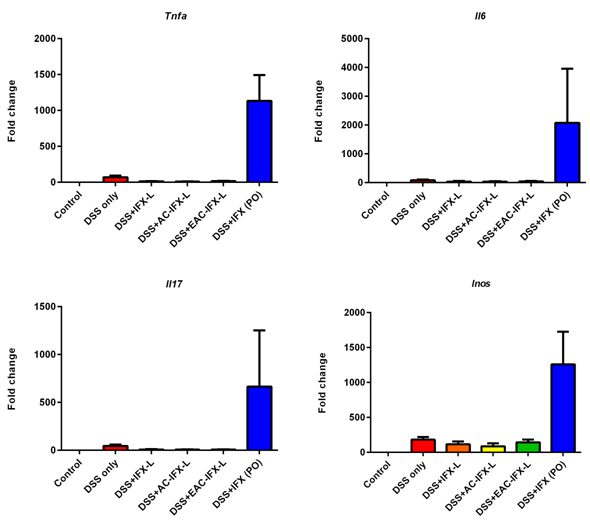
**

**Fig. S4** Comparison of mRNA levels between oral administration of IFX and IFX itself. Gene expression was evaluated by quantitative RT-PCR and was reported as fold change compared to the control by normalizing transcription levels to β-actin. Data are expressed as means ± SD.
